# Correlation between estimated glucose disposal rate and diabetic depression: a population-based study

**DOI:** 10.3389/fpsyt.2025.1507280

**Published:** 2025-03-25

**Authors:** Xiangzhi Shao, Huifang Dai, Lielie Zhu

**Affiliations:** ^1^ Department of Rehabilitation, Wenzhou TCM Hospital of Zhejiang Chinese Medical University, Wenzhou, Zhejiang, China; ^2^ Department of Endocrinology, The Second Affiliated Hospital and Yuying Children’s Hospital of Wenzhou Medical University, Wenzhou, Zhejiang, China

**Keywords:** depression, insulin resistance, waist circumference, diabetes, estimated glucose disposal rate

## Abstract

**Background:**

Emerging evidence has identified a correlation between depression and insulin resistance (IR). This study aims to explore the correlation between estimated glucose disposal rate (eGDR)—a noninvasive and practical measure of IR—and depression in patients with diabetes mellitus (DM).

**Methods:**

In this cross-sectional study, the data from 3,080 adults aged 18 years old or older with DM obtained from NHANES 1999–2018 were analyzed. The correlation between eGDR and depression were examined through multivariate logistic regression, subgroup analyses, restricted cubic spline (RCS) analysis, and interaction tests. Additionally, mediation analysis was conducted to assess whether leukocytes and neutrophils could mediate the effects of eGDR on depression.

**Results:**

Multivariate logistic regression and RCS analyses demonstrate that eGDR was negative linearly correlated with diabetic depression (OR= 0.89; 95% CI: 0.84, 0.95). Patients with DM in Q3 and Q4 of eGDR exhibited a reduced risk of 28% and 54%, respectively, in depression, compared to those in Q1. Subgroup analyses, stratified by variables such as gender, BMI, age, education level, and medical comorbidities, consistently showed a negative correlation. Mediation analysis further indicates that neutrophils and leukocytes accounted for 4.0% and 3.6% of the correlation between eGDR and depression, respectively.

**Conclusions:**

The results of this study demonstrated a statistically significant inverse linear correlation between eGDR and the prevalence of depression in patients with DM, with leukocytes and neutrophils acting as mediating factors in this correlation.

## Introduction

In recent years, diabetes mellitus (DM) has emerged as one of the most common chronic diseases (1). Among those diagnosed with DM, approximately 64% experience psychological distress, while 8% to 35% are also diagnosed with depression (2–5). The coexistence of DM and depression is correlated with poorer adherence to therapeutic regimens and an increased mortality rate (6). Additionally, complications arising from DM can heighten the risk of developing depression. Alarmingly, approximately 51% of depression cases in patients with DM have not yet been diagnosed, and only 31% of those diagnosed have received adequate antidepressant treatment (7). Therefore, the management and identification of depression in patients with DM are both necessary and urgent.

Insulin resistance (IR) has been widely recognized as the primary pathophysiological driver of metabolic syndrome (8, 9). Emerging evidence has highlighted a robust correlation between IR and the development of depression (10, 11), which may be explained by the progression of peripheral IR to central IR, thereby influencing neurobiological processes (10, 11). Central insulin is integral to multiple neural circuits, participating in signal transduction in various glial cells in the brain, modulating dopamine release, and regulating the structure, production, and function of mitochondria. Consequently, these processes collectively impact emotional cognition and behavior (12). Hyperinsulin–hyperglycemic clamp technique is widely regarded as the gold standard in evaluating IR; however, its complexity restricts its practical use in clinical settings (13). As an alternative, eGDR incorporating readily obtainable clinical parameters, such as HbA1C, waist circumference (WC), and hypertension, has been proposed as a more accessible surrogate marker for IR in patients with T1DM (14). Prior investigations have demonstrated that this alternative method has relatively high accuracy to established gold-standard techniques (14, 15). Furthermore, earlier studies have extended the application of eGDR to patients with acute ischemic stroke, T2DM, and non-diabetic conditions, identifying a significant correlation between eGDR and diabetic complications, as well as outcomes, such as CVD mortality and all-cause mortality (16–19). Nonetheless, the correlation between eGDR and depression in patients with DM remains unclear.

It is reasonable to hypothesize a potential correlation between eGDR and the incidence of depression, given the proposition of eGDR serving as an indicator of IR. Consequently, a cross-sectional study was conducted to assess the correlation between eGDR and diabetic depression, aiming to ascertain the predictive value of eGDR for diabetic depression.

## Methods

### Research subjects and design

NHANES, an extensive study aiming to assess the correlation between nutrition, disease prevention, and health promotion administered by NCHS (20), is conducted biennially by taking interviews, physical examinations, with a variety of sections encompassing demographic, dietary, laboratory data, and examination. Further details about the NHANES database can be accessed at http://www.cdc.gov/nhanes.

**Figure 1:**
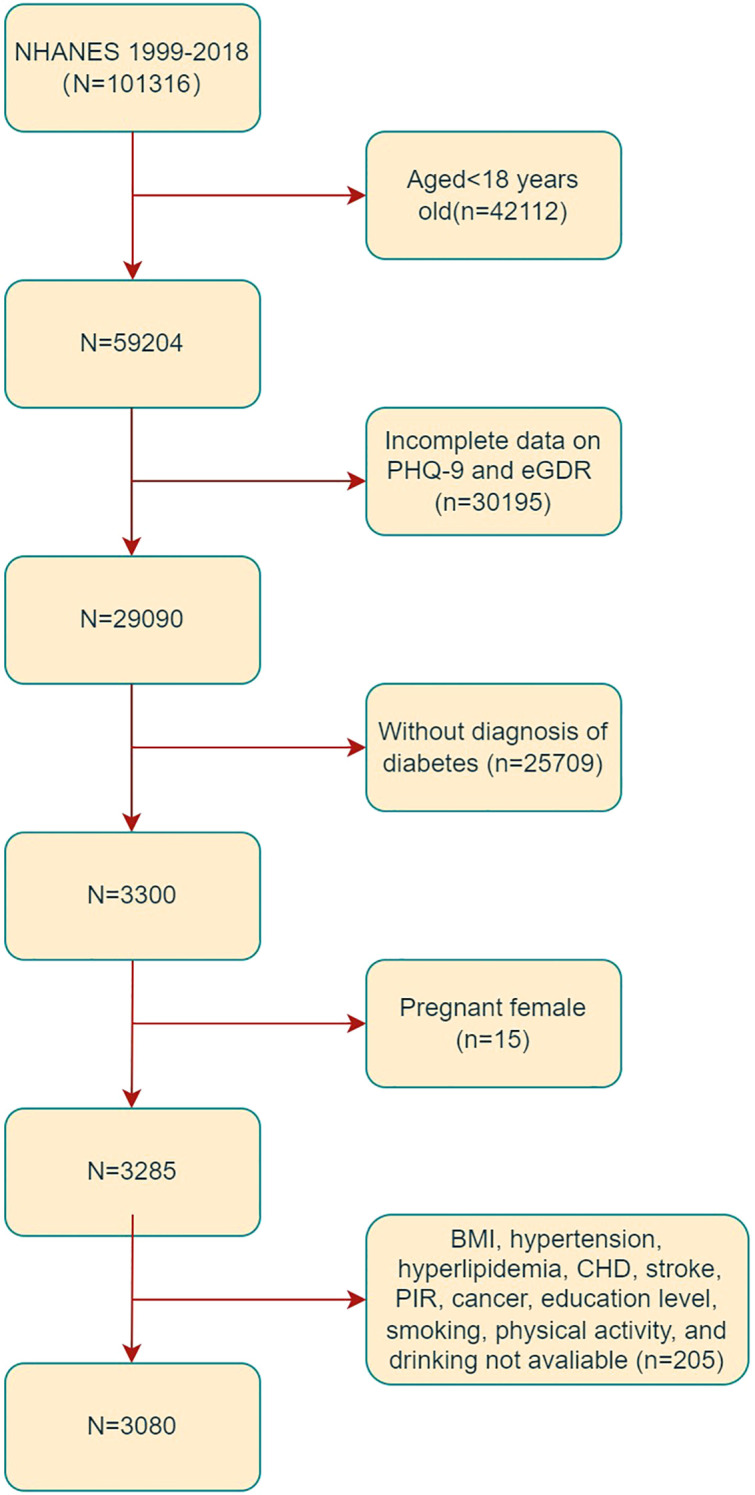
Flowchart of the sample selection from the 1999-2018 NHANES.

In the present study, the data obtained from ten 2-year cycles of NHANES 1999-2018 were analyzed. Subjects aged 18 years old or older were considered eligible for inclusion (n = 59,204). Exclusion criteria were applied to omit subjects with incomplete data on PHQ-9 and eGDR (n =30195), those without a diagnosis of DM (n=25709), and those missing covariate data (n = 205). Additionally, 15 pregnant subjects were excluded from the analysis, as illustrated in **Figure 1**. Consequently, the final study sample comprised 3,080 subjects.

### Ascertainment of depression

PHQ-9 (21) is a well-established tool for screening depression, comprising nine items scored on a 0-3 scale, resulting in a total score ranging from 0 to 27. A total score of 10 or higher is typically used to indicate the presence of depression (21). This cutoff has been extensively validated in clinical settings, which has been widely adopted in epidemiological studies to identify patients with depression (21).

### Assessment of diabetes mellitus

Consistent with the guidelines established by ADA and supported by previous studies (22–24), DM was diagnosed based on the following criteria: (1) HbA1c ≥6.5%; (2) FPG ≥7.0 mmol/L; (3) 2-hour postprandial glucose (2h PG) ≥11.1 mmol/L; (4) a prior diagnosis of DM by a healthcare professional; or (5) the use of hypoglycemic medications or insulin therapy.

### Measurement of eGDR

In this study, eGDR (mg/kg/min) was determined using a previously established formula: eGDR =21.158 - (Hypertension×3.407) - (Waist circumference [cm]×0.09) - (HbA1C [%]×0.551). Hypertension, coded as 0 for absence and 1 for presence (25, 26), was determined based on a physician’s diagnosis, blood pressure readings ≥140/90 mmHg (systolic and/or diastolic), or the current use of antihypertensive medications (27). Subjects were divided into four groups according to eGDR quartiles: <4.28, 4.28–5.96, 5.96–8.60, and ≥8.60, with the lowest eGDR category (<4.28) as the reference group.

### Covariates

The analysis incorporated a range of covariates, encompassing demographic and socioeconomic factors such as gender, poverty-income ratio (PIR), age, marital status, race, and education level. Additionally, lifestyle variables including physical activity levels, smoking status, BMI, and alcohol abuse were considered. The subjects’ medical histories were also accounted for, including conditions such as hyperlipidemia, stroke, hypertension, coronary heart disease (CHD), and various cancers. Furthermore, laboratory test results, including serum leukocyte count, HbA1c, albumin, neutrophil count, and creatinine levels, were also included in the analysis. The definitions for alcohol abuse, smoking, and hyperlipidemia were aligned with those previously documented in the literature (28, 29). eGFR was calculated using the CKD-EPI formula (30).

### Statistical analyses

Subjects included in this study were categorized into two groups based on their PHQ-9 scores: those without depression and those with depression (21). Categorical variables were presented as counts (percentages), whereas continuous variables with normal distribution were reported as means ± SD. The correlation between eGDR and depression was assessed with logistic regression models, to calculate OR and 95% CI. Three models were employed in the analysis: Model 1 was unadjusted; Model 2 was adjusted for gender and age; and Model 3 was the fully adjusted multivariable model, including additional adjustments for physical activities, alcohol abuse, BMI, race, smoking status, stroke, PIR, marital status, CHD, education level, albumin levels, hyperlipidemia, leukocyte count, cancers, and eGFR. The dose–response correlation between depression and eGDR was examined with RCS curves, with a focus on detecting the potential non-linear correlation. Additionally, subgroup analyses were conducted to assess whether the correlation between eGDR and depression differed across patients with varying characteristics. Mediation analyses were then conducted with the mediation package, and the confidence interval for the mediation effect was assessed to quantify the contributions of leukocytes and neutrophils. The data analyses were conducted with R software and Free Statistics software, with statistical significance set at a two-sided P value less than 0.05.

## Results

### Baseline characteristics

**Table 1** provides a comprehensive overview of the characteristics of the research subjects. Among the 3,080 subjects, 1,575 (51.1%) were females, with a mean age of 58.5 ± 17.2 years old. Depression was identified in 352 subjects (11.4%). Patients with depression were predominantly female, lived alone, and had a higher prevalence of recent or past smoking. They also exhibited lower levels of family PIR and albumin. Additionally, these individuals were more likely to engage in insufficient physical activities, with higher levels of BMI, WC, HbA1c, leukocytes, and neutrophils. They were also more frequently correlated with underlying medical conditions such as stroke, CHD, and hypertension. The mean eGDR was 5.73 ± 2.95 in the depression group, which was lower than 6.40 ± 2.83 observed in the non-depression group.

**Table 1:** Characteristics of the study population based on depression.

Characteristic	Total (n=3080)	PHQ-9<10 (n=2728)	PHQ-9≥10 (n=352)	P value
Age	58.5 ± 17.2	58.8 ± 17.2	55.9 ± 16.3	0.003
Gender, %				< 0.001
Male	1505 (48.9)	1376 (50.4)	129 (36.6)	
Female	1575 (51.1)	1352 (49.6)	223 (63.4)	
Race, %				0.145
Mexican American	522 (16.9)	452 (16.6)	70 (19.9)	
Other Hispanic	296 (9.6)	257 (9.4)	39 (11.1)	
Non-Hispanic White	1270 (41.2)	1128 (41.3)	142 (40.3)	
Non-Hispanic Black	751 (24.4)	668 (24.5)	83 (23.6)	
Other Race	241 (7.8)	223 (8.2)	18 (5.1)	
Education level, %				< 0.001
Less than high school	1031 (33.5)	872 (32)	159 (45.2)	
High school or above	2049 (66.5)	1856 (68)	193 (54.8)	
Marital, %				0.008
Married/living with partner	1837 (59.6)	1653 (60.6)	184 (52.3)	
Separated/divorced/widowed	856 (27.8)	745 (27.3)	111 (31.5)	
Never married	387 (12.6)	330 (12.1)	57 (16.2)	
Moderate physical activity, %				< 0.001
Yes	2091 (67.9)	1800 (66.0)	291 (82.7)	
No	989 (32.1)	928 (34.0)	61 (17.3)	
Alcohol status, n%				0.557
Current or ever, %	2075 (67.4)	1833 (67.2)	242 (68.8)	
Never	1005 (32.6)	895 (32.8)	110 (31.2)	
Smoking status, n%				< 0.001
Current or ever, %	1574 (51.1)	1354 (49.6)	220 (62.5)	
Never	1506 (48.9)	1374 (50.4)	132 (37.5)	
Hypertension, %				0.010
Yes	1818 (59.0)	1588 (58.2)	230 (65.3)	
No	1262 (41.0)	1140 (41.8)	122 (34.7)	
Hyperlipidemia, %				0.214
Yes	2433 (79.0)	2146 (78.7)	287 (81.5)	
No	647 (21.0)	582 (21.3)	65 (18.5)	
CHD, %				0.007
Yes	282 (9.2)	236 (8.7)	46 (13.1)	
No	2798 (90.8)	2492 (91.3)	306 (86.9)	
Stroke				<0.001
Yes	242 (7.9)	198 (7.3)	44 (12.5)	
No	2838 (92.1)	2530 (92.7)	308 (87.5)	
Cancer, %				0.587
Yes	409 (13.3)	359 (13.2)	50 (14.2)	
No	2671 (86.7)	2369 (86.8)	302 (85.8)	
Body mass index, kg/m^2^	30.1 ± 7.1	29.9 ± 6.9	31.6 ± 8.5	< 0.001
Waist circumference, cm	103.3 ± 17.0	103.0 ± 16.7	106.1 ± 19.1	0.001
HbA1c, %	6.40 ± 1.70	6.37 ± 1.66	6.63 ± 1.96	0.006
Albumin, g/dl	41.6 ± 3.6	41.7 ± 3.5	40.9 ± 4.1	< 0.001
Leukocyte, 10^9^/L	7.54 ± 2.81	7.49 ± 2.86	7.91 ± 2.41	0.009
Neutrophil, 10^9^/L	4.58 ± 2.10	4.54 ± 2.11	4.90 ± 1.98	0.002
Creatinine, umol/L	79.6 (63.6, 102.5)	79.6 (63.6, 103.4)	77.8 (62.1, 100.6)	0.138
eGFR, ml/min/1.73m^2^	79.4 (60.2, 99.9)	79.6 (60.3, 99.4)	76.5 (59.5, 104.6)	0.977
PIR	2.19 ± 1.50	2.28 ± 1.52	1.47 ± 1.14	< 0.001
eGDR, mg/kg/min	6.32 ± 2.85	6.40 ± 2.83	5.73 ± 2.95	< 0.001

Values are mean±SD or number (%). P<0.05 was deemed significant. PIR, poverty-income ratio; eGFR, eGDR, estimated glucose disposal rate; CHD, coronary heart disease.

### Correlation between eGDR and depression in subjects with diabetes mellitus

**Figure 2:**
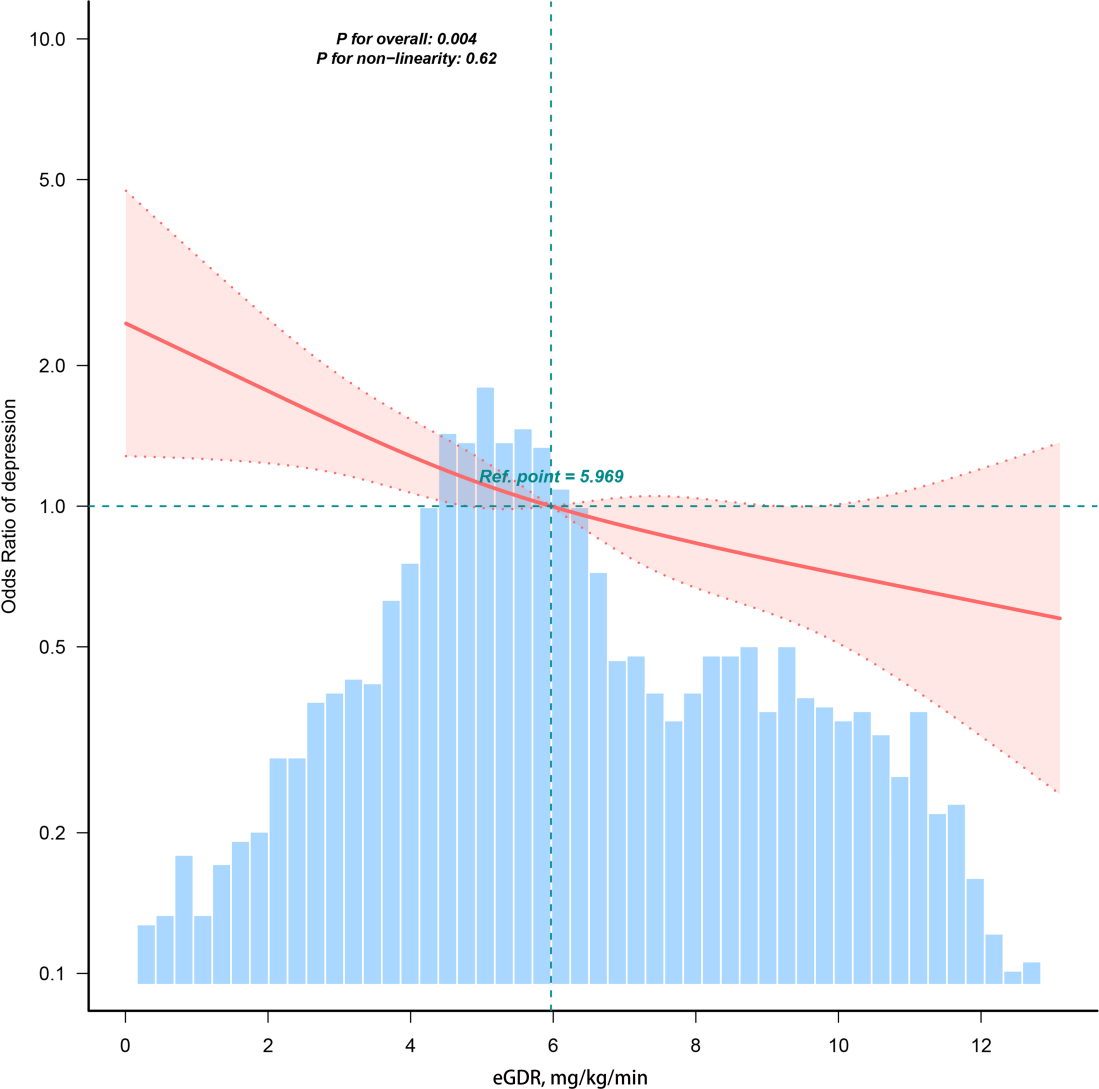
Restricted cubic spline fitting for the association between eGDR and depression. Ref represents the median of the eGDR values.

In the multiple logistic regression analysis, a significant inverse correlation was identified between depression and eGDR after adjusting for confounding variables in Model 3 (OR=0.89, 95%CI: 0.84, 0.95). To further explore this correlation, subjects were categorized into quartiles based on eGDR values. In the fully adjusted Model 3, subjects in the third and fourth quartiles of eGDR exhibited ORs of 0.72 (95% CI: 0.55–0.99) and 0.46 (95% CI: 0.28–0.74), respectively, compared to those in the first quartile (reference group) for the risk of developing depression (p = 0.003 for trend); these results are detailed in **Table 2**. RCS analyses revealed a linear correlation between eGDR and depression, as depicted in **Figure 2**.

**Table 2:** Associations between eGDR and depression.

subgroups	Model1		Model2		Model3	
	OR (95%CI)	P-value	OR (95%CI)	P-value	OR (95%CI)	P-value
eGDR	0.92 (0.88, 0.96)	<0.001	0.89 (0.85, 0.92)	<0.001	0.89 (0.84, 0.95)	<0.001
eGDR (category)						
Q1	1(Ref)		1(Ref)		1(Ref)	
Q2	0.74 (0.55, 1.00)	0.050	0.76 (0.56, 1.03)	0.074	0.76 (0.55, 1.06)	0.111
Q3	0.73 (0.54, 0.99)	0.042	0.68 (0.50, 0.92)	0.013	0.72 (0.55, 0.99)	0.048
Q4	0.51 (0.37, 0.71)	<0.001	0.38 (0.27, 0.53)	<0.001	0.46 (0.28, 0.74)	0.001
P for trend	0.82 (0.74, 0.91)	<0.001	0.75 (0.67, 0.83)	<0.001	0.80 (0.69, 0.93)	0.003

Model 1, None covariates were adjusted; Model 2, gender and age were adjusted; Model 3, gender, age, race, alcohol consumption, BMI, smoking, moderate physical activities, CHD, stroke, PIR, education level, marital status, albumin, hyperlipidemia, leukocyte, eGFR and cancers were adjusted.

### Subgroup analysis

**Figure 3:**
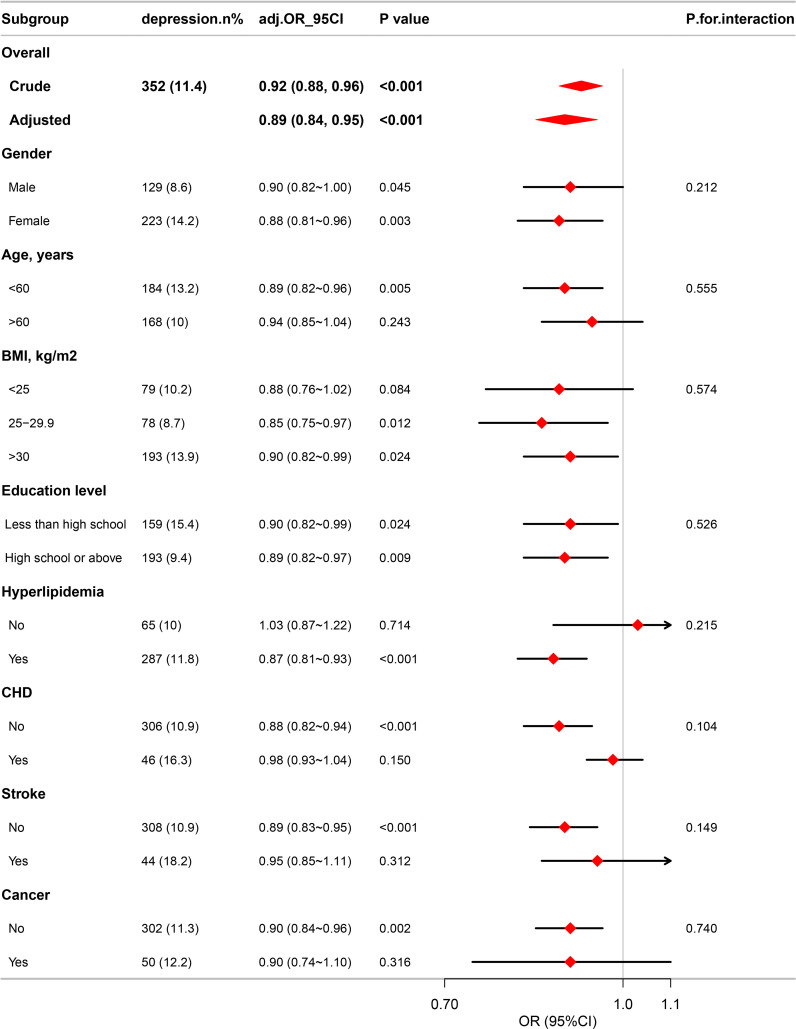
Association between eGDR and the risk of depression in various subgroups.

Subgroup analyses were conducted to further explore the correlation between eGDR and depression in diverse population groups stratified by BMI, age, education level, gender, and disease status, including CHD, hyperlipidemia, cancers, and stroke. The analyses revealed no significant differences across all subgroups, as illustrated in **Figure 3**.

### Mediation analysis

**Figure 4:**
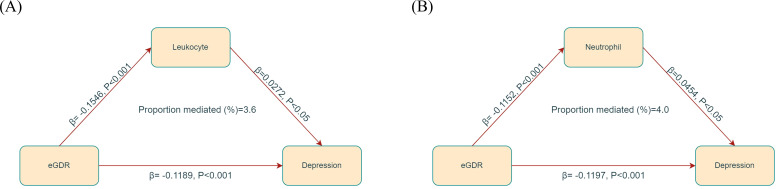
Mediated analysis model path diagram. eGDR was defined as the independent variable; depression as the dependent variable; and leukocyte **(A)** and neutrophil **(B)** as the mediating variable.

In the mediation analysis, eGDR was assigned as the independent variable, with leukocytes and neutrophils functioning as mediators, and depression being the dependent variable. As illustrated in **Figure 4**, leukocytes and neutrophils contributed to 3.6% and 4.0% of mediation effects in the correlation between eGDR and depression, respectively. A detailed summary of the mediation analysis outcomes, encompassing mediation ratios, direct effects, total effects, and indirect effects, is presented in **Table 3**.

**Table 3:** Mediation analysis of leukocyte, neutrophil in the association between eGDR and depression.

Independent variable	Mediator	Total effect		Indirect effect		Direct effect		Proportion mediated, %
		Coefficient (95% CI)	P value	Coefficient (95% CI)	P value	Coefficient (95% CI)	P value	
eGDR	leukocyte	-0.0195(-0.0295, -0.0111)	<0.001	-0.0007(-0.0017, -0.0)	0.048	-0.0189 (-0.0289, -0.0105)	<0.001	3.6
eGDR	neutrophil	-0.0199(-0.0291, -0.011)	<0.001	-0.0008(-0.0022, -0.0001)	0.032	-0.019(-0.028, -0.01)	<0.001	4.0

## Discussion

This study identified a negative correlation between depression and eGDR in patients with DM. This correlation remains consistent irrespective of education level, age, BMI, gender, and the presence of medical comorbidities. Additionally, mediation analysis revealed that leukocytes and neutrophils can partially mediate the correlation between depression and eGDR.

Numerous prior studies have established a significant correlation between depression and IR. Notably, an in-depth analysis on the data from the Netherlands Study identified a correlation between IR and persistent, chronic major depressive disorder, suggesting that IR may be a distinctive characteristic of depressive disorders (31). Furthermore, Adriaanse et al. conducted a study involving 541 subjects aged 55 to 75 years old in Netherlands, demonstrating a weak correlation between IR and depression, as measured by HOMA-IR (32). Additionally, a cross-sectional study in South Korea, encompassing over 160,000 subjects, indicates that an increased risk of developing depression is correlated with IR. Specifically, as IR increases, the risk of developing depression rises by 4% in young adults and 17% in non-diabetic individuals (33).

In conjunction with readily accessible clinical parameters such as WC, hypertension, and HbA1c, eGDR has been proposed as a simple surrogate marker for IR in patients with T1DM (14). Previous studies have demonstrated that this alternative method exhibits high accuracy when comparing with established gold standards (14, 15). Moreover, eGDR has been identified as an independent predictor of CHD (34), peripheral vascular disease (35), and all-cause mortality (36) in patients with T1DM. In recent years, the applicability of eGDR has been explored in patients without DM (37), those with T2DM (15, 38), and those with acute ischemic stroke (15, 39). Given that many of these conditions are correlated with vascular damage and sclerosis, which are significant risk factors for developing depression, a strong correlation between eGDR and depression in patients with DM was hypothesized. To the best of our knowledge, this study is the first to explore the correlation between eGDR and depression in patients with DM.

The exact mechanisms underlying the correlation between IR and depression remain incompletely understood. Nevertheless, multiple potential mechanisms have been hypothesized. Accumulating data indicate that patients with depression often display dysfunction in homeostatic systems, particularly in inflammatory responses and hypothalamic-pituitary-adrenal (HPA) axis. These systems have been implicated in the development of IR and MetS. Dysregulation of the HPA axis has been linked to the onset of depression (40, 41), with changes in glucocorticoid sensitivity and systemic cortisol effects observed in patients with stress-related disorders (42). Overactivation of the HPA axis is correlated with increased visceral adiposity, elevated lipid storage, and enhanced lipogenesis. Moreover, in severe obesity, visceral adipose tissues can function as an endocrine organ, secreting various hormones and inflammatory cytokines (43, 44). These cytokines are capable of traversing the blood-brain barrier, thereby disrupting neurotransmission and inducing neurological dysfunction, while also inhibiting neurogenesis in brain regions implicated in mood regulation (45). The elements constituting eGDR, including waist hypertension, WC, and HbA1c, play a crucial role in these processes. Consequently, examining these mechanisms can elucidate potential pathways linking eGDR, a non-insulin-based indicator of insulin resistance, with depression.

The findings indicate that inflammation, as assessed by leukocyte and neutrophil counts, can partially mediate the correlation between IR, measured by eGDR, and depression, highlighting the critical need to monitor inflammation levels in patients with DM exhibiting low eGDR. IR can induce oxidative stress and exacerbate inflammatory responses, as evidenced by increased leukocyte and neutrophil levels (46, 47). Previous research has established a significant link between depression and chronic inflammation (48, 49). Consequently, managing chronic inflammation, particularly through the reduction of leukocyte and neutrophil levels, may reduce the risk of developing depression in patients with DM exhibiting low eGDR.

This study demonstrated several notable strengths. Its sample size was sufficiently large to detect a significant correlation between depression and eGDR in patients with DM. Moreover, the study was meticulously controlled for a wide range of confounding variables, yielding robust estimates of the independent correlation between depression and eGDR. Furthermore, an intermediary analysis was conducted to explore the correlation between IR, inflammation, and depression.

However, several limitations of this study must be acknowledged. Firstly, the cross-sectional nature of the study designed limits the ability to establish a causal correlation between eGDR and depression. Future research should employ longitudinal studies and experimental approaches to clarify the temporal correlation and underlying mechanisms. Secondly, the diagnosis of depression with PHQ-9 relies predominantly on self-reported data, which may not be directly validated by clinical professionals. However, it is important to note that PHQ-9 has been widely used in clinical and epidemiological contexts and has undergone extensive validation (50). Thirdly, the study’s findings pertain specifically to American patients with DM, which may constrain the generalizability of the results to other demographic groups. Furthermore, the study is constrained by its data time span (1999–2018), which may not adequately reflect clinical practice or social changes occurring after 2018. Additionally, due to insufficient information, there may be unmeasured confounding factors that could not be adjusted for, such as the use of antidepressants, insulin therapy, and the duration of DM. Consequently, comprehensive longitudinal prospective studies with detailed data collection are essential in the future, to validate the correlation between eGDR and depression.

## Conclusion

In summary, this study revealed a significant negative correlation between eGDR and depression in patients with DM. This independent correlation indicates that eGDR can potentially function as a valuable biomarker for screening depression.

## Data Availability

Publicly available datasets were analyzed in this study. This data can be found here: NHANES, http://www.cdc.gov/nhanes.
